# Identification of a Novel Homozygous Mutation, *TMPRSS3: c.535G>A*, in a Tibetan Family with Autosomal Recessive Non-Syndromic Hearing Loss

**DOI:** 10.1371/journal.pone.0114136

**Published:** 2014-12-04

**Authors:** Dongyan Fan, Wei Zhu, Dejun Li, De Ji, Ping Wang

**Affiliations:** 1 Department of Otolaryngology-Head and Neck Surgery, the First Hospital of Jilin University, Changchun, Jilin Province, China; 2 Tibet University School of Medicine, Lhasa, China; 3 Center for Prenatal Diagnosis, the First Hospital of Jilin University, Changchun, Jilin Province, China; Oslo University Hospital, Norway

## Abstract

Different ethnic groups have distinct mutation spectrums associated with inheritable deafness. In order to identify the mutations responsible for congenital hearing loss in the Tibetan population, mutation screening for 98 deafness-related genes by microarray and massively parallel sequencing of captured target exons was conducted in one Tibetan family with familiar hearing loss. A homozygous mutation, *TMPRSS3: c.535G>A,* was identified in two affected brothers. Both parents are heterozygotes and an unaffected sister carries wild type alleles. The same mutation was not detected in 101 control Tibetan individuals. This missense mutation results in an amino acid change (p.Ala179Thr) at a highly conserved site in the scavenger receptor cysteine rich (SRCR) domain of the TMPRSS3 protein, which is essential for protein-protein interactions. Thus, this mutation likely affects the interactions of this transmembrane protein with extracellular molecules. According to our bioinformatic analyses, the *TMPRSS3: c.535G>A* mutation might damage protein function and lead to hearing loss. These data suggest that the homozygous mutation *TMPRSS3: c.535G>A* causes prelingual hearing loss in this Tibetan family. This is the first *TMPRSS3* mutation found in the Chinese Tibetan population.

## Introduction

Hearing impairment is a common birth defect. Epidemiologic studies have shown that 50% of childhood deafness cases are associated with genetic defects. Hearing loss caused by genetic defects is also characterized by high allelic and locus heterogeneity. Many deafness genes, including the well-known deafness genes *GJB2* (MIM#121011), *SLC26A4* (MIM#605646) and *12S rRNA* (MIM#561000), have been identified [1]–[5]. In the Chinese deaf population, 21% have *GJB2* mutations, 14.5% have *SLC26A4* mutations, and 3.4% have the *12S rRNA:A1555G* mutation [2]. High genetic heterogeneity suggests that hearing loss may be caused by variable mutations in hundreds of genes or that a deafness mutation may lead to different deafness phenotypes. Moreover, people with different ethnicities may have different mutation spectrums. Li *et al.* reported that different ethnic groups present different incidences of large vestibular aqueduct (LVA) disorders [6]. In China, Han Chinese has the highest incidence of LVA disorders, while minorities in southwest China have low incidences. In our previous study, two cases with heterozygous *GJB2* mutations and two cases with homoplasmic *12S rRNA:A1555G* mutations were identified from 92 Tibetan prelingual deafness students who underwent mutation screening for *GJB2*, *SLC26A4*, and mitochondria DNA *12S rRNA* mutations [7]. These studies suggested that deafness mutation hotspots are variable in populations with different ethnicities. Consistent with those findings, Dai *et al.* showed that only the *12S rRNA:A1555G* mutation was detected in four individuals from 114 Tibetan deafness patients [5]. They suggested that the different mutation spectrums in Han Chinese and Tibetan Chinese might have been related to distinct geographic features in their living areas. Tibetan Chinese live in high altitude regions, which may contribute to the increased incidence of deafness in Tibetan Chinese due to the low oxygen micro-environment in the inner ear.

The *TMPRSS3* (Transmembrane protease, serine 3, MIM#605511) gene encodes an enzyme that is expressed in multiple tissues, including inner hair cells, spiral ganglion neurons, and stria vascularis of the cochlear duct. *TMPRSS3* is involved in the development and maintenance of the inner ear perilymph and endolymph [8], [9]. Many mutant alleles of *TMPRSS3* have been reported to be associated with nonsyndromic recessive deafness (DFNB8/B10) [8], [10], [11]. Although *TMPRSS3* mutations seem to be less common than *GJB2* mutations, *TMPRSS3* mutations lead to inheritable deafness, especially when common *GJB2*, *SLC26A4 and 12S rRNA* mutations are excluded.

The TMPRSS3 protein contains several common functional domains, including a transmembrane domain, a low density lipoprotein receptor A domain (LDLRA), a scavenger receptor cysteine rich domain (SRCR), and a carboxyl terminal serine protease domain [9]. Many *TMPRSS3* mutations have been reported as pathogenic mutations for inheritable deafness, including an 8-bp deletion, insertion of multiple beta-satellite repeat units, and a frameshift mutation [8].

In order to explore the distinct mutation spectrum associated with inheritable deafness in Tibetan Chinese, we performed mutation screening on 98 deafness genes using both microarray and massively parallel sequencing of captured exons in an affected Tibetan family. We defined the homozygous mutation of *TMPRSS3: c.535G>A* as the genetic etiology for two brothers. To our knowledge, this is the first report of a *TMPRSS3* mutation causing deafness in Chinese.

## Materials and Methods

### Ethics Statement

The experimental protocol was established according to the ethical guidelines of the Helsinki Declaration and was approved by the Ethics Committee of the First Hospital of Jilin University in China. Written informed consents were obtained from all of the adult participants. If the participant was underage, the participants’ parents or guardians signed the consent.

### Subjects

The proband, a 12-year old boy living at an altitude of 3560 meters, failed otoacoustic emission (OAE) tests bilaterally. His auditory brainstem response (ABR) test showed response threshold values more than 85 dBnHL bilaterally. These test results suggested profound sensorineural hearing loss. The proband’s 10-year old brother was also diagnosed with profound bilateral sensorineural hearing loss (BSNHL). General physical checkups and examinations for overall development were normal. In addition, eye and thyroid gland tests showed normal results, excluding the possibility of syndromic hearing loss. Deafness caused by otitis media, meningitis, viral encephalitis, parotitis, and general infection were also excluded for both siblings. The parents and sister have normal hearing. Parents claimed no familiar history of hearing loss or consanguinity.

### DNA Sample Collection

Peripheral blood specimens (5 mL) were collected from each individual for DNA analysis. Genomic DNA was extracted using a TIANamp Blood DNA Kit (Tiangen Biotech, Beijing, China). Genomic DNA products (concentration = 100∼150 ng/µl; OD260/OD280 = 1.7∼1.9) were purified according to manufacturer’s instructions and stored in a −20°C freezer.

### Mutation Detection by Microarray

A Deafness Gene Mutation Detection Array kit (CapitaBio, Beijing, China) was used to detect the following known hotspot mutations related to hereditary hearing loss: *GJB2* (35 delG, 176 del16, 235 delC, and 299–300 delAT), *GJB3* (538C>T) (MIM#603342), *SLC26A4* (IVS7-2A>G and 2168A>G), and *12S rRNA* (1494C>T and 1555A>G). The standard procedures recommended by the manufacturer were applied to detect nine mutations in the four genes. Target DNA fragments were amplified with nine pairs of primers in two multiplex PCR reactions with the following components: 150 ng of template genomic DNA, 1.5 µl of 10x Hotstart buffer, 2.5 mM dNTP, primer mix, 5 u/µl of HotStart HiFidelity DNA Polymerase and ddH_2_O in 15 µl of total volume. PCR reactions were run with the following conditions: initial incubation at 95°C for 15 min and at 96°C for 1 min, followed by 32 cycles of 94°C for 30 sec, 55°C for 30 sec, and 70°C for 45 sec. A final incubation step was performed at 60°C for 10 mins. Hybridization reactions were performed according to the manufacturer’s manual using a BioMixerTM II Microarray Hybridization Station (CapitaBio, Beijing, China). A LuxScanTM 10K Microarray Scanner (CapitaBio, Beijing, China) was used for microarray data capture.

### Illumina Library Preparation

Sample DNA was extracted from patient blood using the DNA Extraction kit (DP319, Tiangen, Beijing, China). The DNA was first quantified with a Nanodrop 2000 Spectrophotometer (Thermal Fisher Scientific, DE). A minimum of 3 µg of DNA was used to create the indexed Illumina libraries according to the manufacturer’s protocol. The final library, which had a size ranging from 300 bp to 400 bp including adapter sequences, was finally selected.

### Deaf Panel Gene Enrichment and Sequencing

All of the exons of the 98 deafness genes were enriched using the GenCap Human Deafness Genes enrichment kit (MyGenostics Inc., Beijing, China). The biotinylated 60-mer oligo baits were designed to tile all of the exons. The capture experiment was conducted according to the manufacturer’s protocol. In brief, 1 µg of DNA library was mixed with Buffer BL and biotinylated probes (MyGenostics Inc., Beijing, China) and then heated at 95°C for 7 min and 65°C for 2 min on a PCR machine. Next, 23 µl of the 65°C pre-warmed Buffer HY (MyGenostics Inc., Beijing, China) was added to the mixture, which was subsequently held at 65°C for 22 hours for hybridization. Next, 50 µl of MyOne beads (Life Technology, USA) were washed in 500 µL of 1X binding buffer three times and resuspended in 80 µl of 1X binding buffer. Then, 64 µl of 2X binding buffer was added to the hybrid mixture and transferred to the tube containing 80 µl of MyOne beads. The mixture was rotated for 1 hour on a rotator. The beads were then washed once with WB1 buffer at room temperature for 15 min and washed three times with WB3 buffer at 65°C for 15 min. The bound DNA was then eluted with Buffer Elute. The eluted DNA was finally amplified using the following conditions: 98°C for 30 sec (1 cycle); 98°C for 25 sec, 65°C for 30 sec, 72°C for 30 sec (15 cycles); and 72°C for 5 min (1 cycle). The PCR product was purified using SPRI beads (Beckman Coulter) according to the manufacturer’s protocol. The enrichment libraries were sequenced on an Illumina HiSeq 2000 sequencer for 100 bp paired reads.

### Confirmation by Sanger Sequencing

The following primers were designed to amplify exon 6 of *TMPRSS3*: TMPRSS3-ex6-Forward, 5′-TCTCCCACCATCTTCCTA-3′; TMPRSS3-ex6-Reverse, 5′-ACTGATGCCAACACCAAC-3′. PCR amplification was performed with the following conditions: 95°C for 15 min; 96°C for 1 min; 32 cycles of 94°C for 30 sec, 55°C for 30 sec, and 72°C for 45 sec; 72°C for 5 min. Purified PCR products with a size of 420 bp were sent for Sanger sequencing (Sangon Biotech, Shanghai, China). Sequencing results were analyzed and aligned against the *TMPRSS3 NM_032404* sequence shown in the NCBI database using the GeneTool 1.0 software (BioTools Inc.). Sequencing data from 101 normal Tibetan children were used as control.

### Data Analysis

The SOAPaligner software was used for sequence alignment and data quality analysis (BGI, China). The SOAPsnp software was used for SNP genotyping (BGI, China). SNP data with poor quality (quality value<20) and low coverage (depth<10) were filtered. Annotations for SNP variants were completed using multiple databases, including Consensus CDS (CCDS) database, human genome builder NCBI 36.3, and dbSNP database V130. For all of the variants, the results were filtered using a quality value of single base sequencing ≧20. Online tools, SIFT, PolyPhen-2, and MutationTaster were used to predict the functional outcome of the detected SNP variants.

## Results

### Audiology Tests

**Figure 1:**
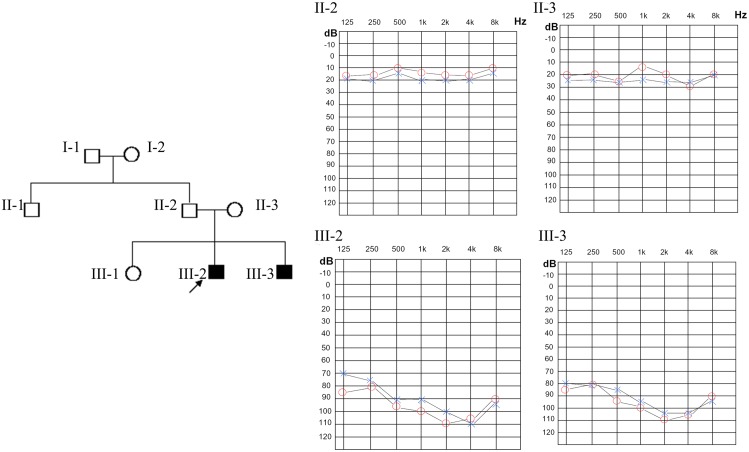
Family pedigree and relative audiograms. Audiograms of family members were obtained using pure tone audiometery with air conduction at frequencies from 250 to 8000 Hz. The proband’s parents, II-2 at age 41 and II-3 at age 39, showed normal hearing audiograms, while the affected brothers, III-2 and III-3, showed severe to profound hearing loss.

Family pedigree and audiograms are shown in Figure 1. Pure tone audiology examinations showed that both brothers, III-2 and III-3 in the pedigree, had profound BSNHL. They both had a 70∼90 decibels hearing level (dB HL) at a frequency spectrum from 0.5 to 2.0 kHz. The hearing curve was flat and showed decreased hearing at all frequencies. The family members, II-1, II-2 and II-3, have normal hearing. III-2 and III-3 showed type A tympanograms (data not shown). Stapedial acoustic reflex and otoacoustic emission (OAE) were not detected at all frequencies (data not shown).

### Detection of Deafness Genes Mutations by Microarray

Mutations were not detected for the brothers with BSNHL by the microarray assay. These results ruled out the nine common deafness mutations of *GJB2*, *GJB3*, *SLC26A4*, and *12S rRNA* in the two affected brothers.

### Identification of Deafness Mutations by Target Capture and Massively Parallel Sequencing

**Figure 2:**
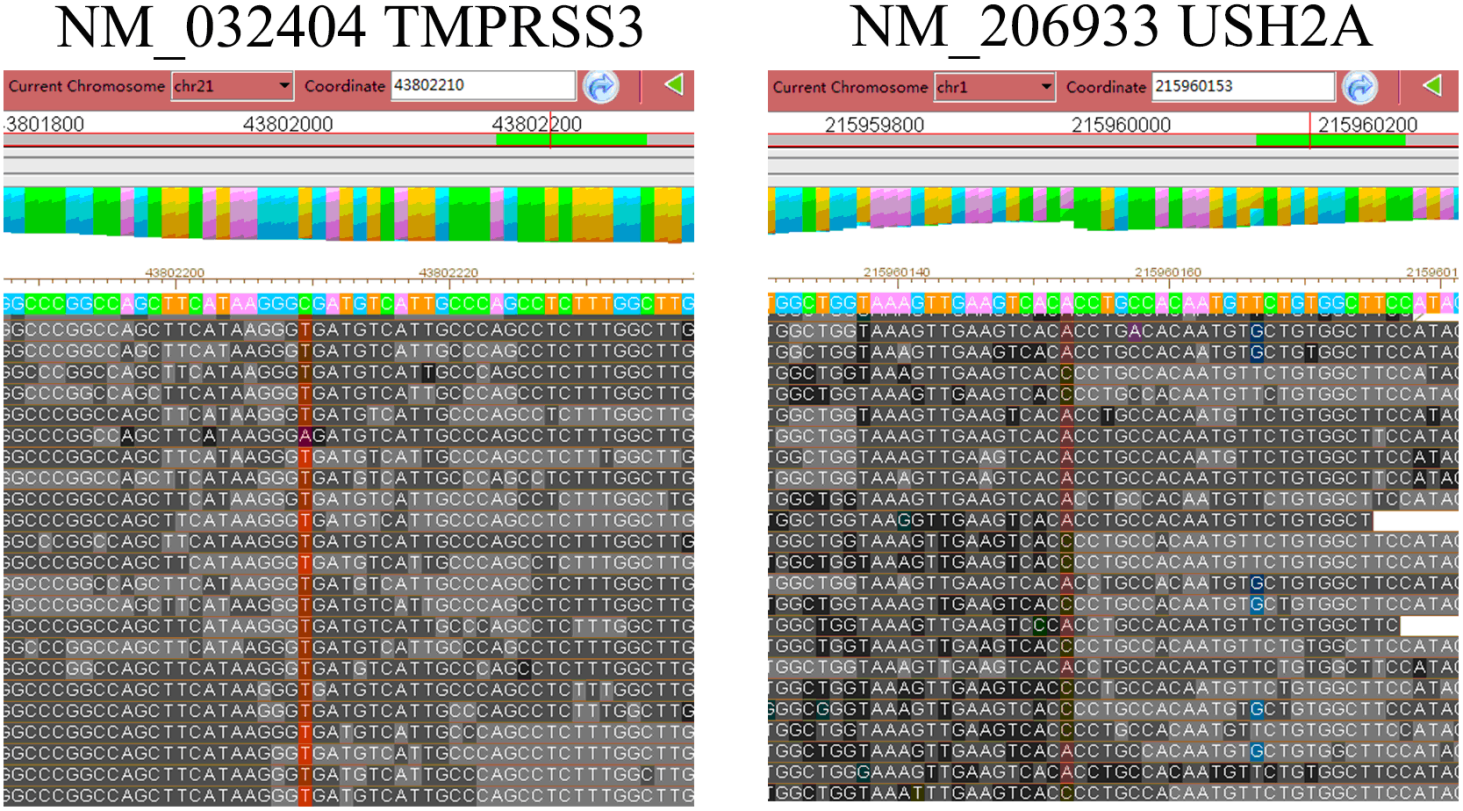
Deep sequencing data for the homozygous mutation in *TMPRSS3* and the heterozygous mutation in *USH2A*. Sequencing alignment data for the homozygous mutation *TMPRSS3: c.535G>A* is shown in the left panel. Sequencing alignment data for the heterozygous mutation *USH2A: c.10246 T>G* is shown in the right panel.

Thereafter, deep sequencing data for exons of 98 deafness genes were analyzed. Overall, 98.0% of the targeted disease gene regions were sequenced and 94.1% of the targeted bases were sequenced with >10X depth, allowing us to accurately assess SNPs. In total, 188 variants were identified in the proband’s sample. Of the 188 variants, 96 were non-synonymous, missense, nonsense or splice variants. These were narrowed to three variants by excluding variants reported in the HapMap 28 and the SNP release of the 1000 Genome Project with a minor allele frequency >0.01. Further analyses showed that the proband III-2 had a homozygous mutation in exon 6 of *TMPRSS3* (*TMPRSS3: c.535G>A*) (Table 1 and Figure 2). This proband also carried a heterozygous mutation in *USH2A* (*USH2A:c.10246T>G*) (MIM#608400).

**Table 1:** Candidate SNPs identified in the proband with BSNHL.

Gene Symbol	Transcript	Exon	Nucleotide Change	Amino Acid Change	Position	Mode
TMPRSS3	NM_032404	exon6	c.535G>A	p.A179T	chr21–43802210	Hom.
USH2A	NM_206933	exon2	c.10246T>G	p.C3416G	chr1–215960153	Het.

### Mutation Confirmation by Sanger Sequencing

**Figure 3:**
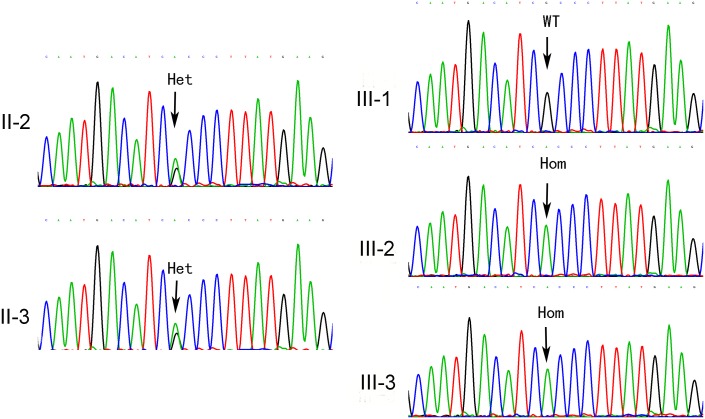
Data from Sanger sequencing for the two affected patients and the direct relatives in the family. Homozygous mutation genotype A/A in the two affected brothers, III-2 and III-3. Wild type genotype G/G in the unaffected sister, III-1. Heterozygous genotype G/A in both parents, II-2 and II-3.

Sanger sequencing for the affected brothers and their direct relatives confirmed the mutations in the brothers. In addition, Sanger sequencing revealed that the parents, II-2 and II-3, are heterozygous carriers for the mutation *TMPRSS3: c.535G>A*. In addition, the normal sister, III-1, has two wild type alleles at this locus (Figure 3).

### Functional Outcome Predicted by SIFT, Polyphen-2, and Mutationtaster

**Figure 4:**
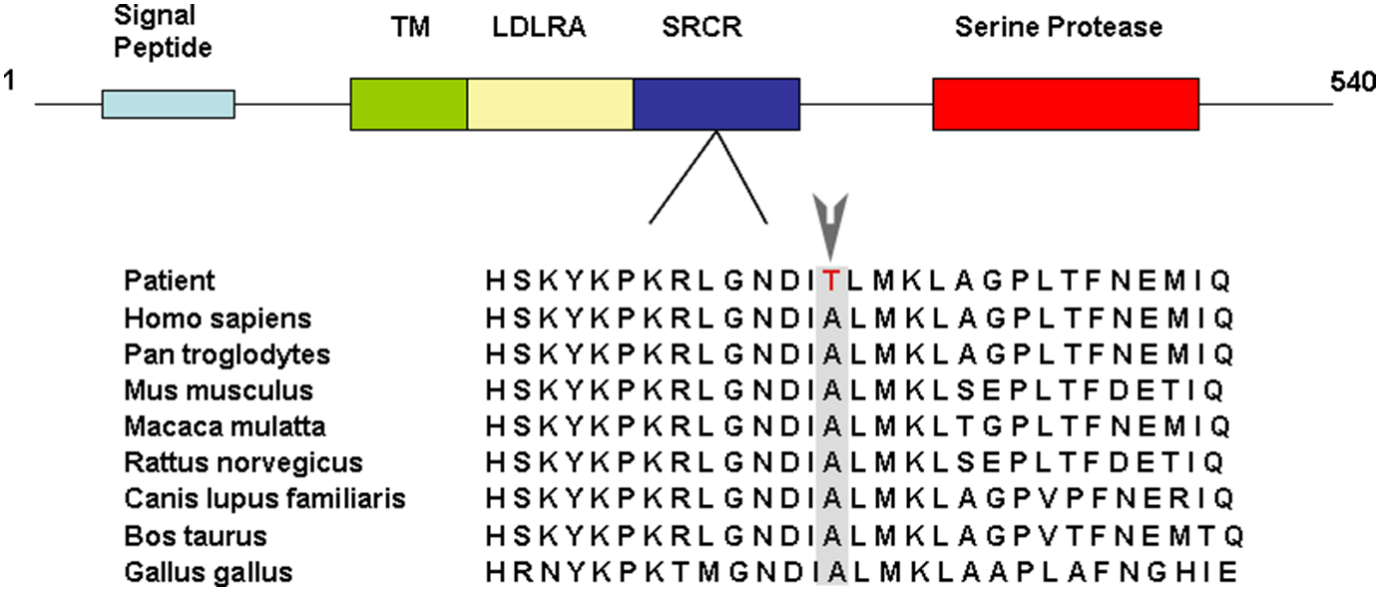
Human TMPRSS3 protein domains and the analysis of evolutionary conservation for the sequence around the mutation site. The TMPRSS3 protein consists of 540 amino acids and contains a signal peptide, a transmembrane (TM) domain, a low density lipoprotein receptor A (LDLRA) domain, a scavenger receptor cysteine rich (SRCR) domain, and a carboxyl terminal serine protease domain. The Alanine (179) in the SRCR domain, which is mutated to Threonine in the two patients, is highly conserved in vertebrates.

The *TMPRSS3: c.535G>A* mutation identified in this family is a missense mutation and leads to an amino acid change from Ala to Thr (p.Ala179Thr). Alanine (A) is a highly conserved amino acid in vertebrate species (Figure 4). This mutation was predicted to “affect protein function” by SIFT, “probably damaging” by PolyPhen-2, and “disease causing” by MutationTaster (Table 2).

**Table 2:** Functional prediction for the *TMPRSS3* missense mutation, c.535G>A.

Algorithm	c.535G>A (p.A179T)
PolyPhen-2	Probably damaging (Score: 0.99)
SIFT	Affect protein function (Score:0.00)
MutationTaster	Disease causing

## Discussion

In our study, new mutation detection techniques such as microarray and massive parallel sequencing of captured target exons were used to screen 98 known deafness genes in Tibetan patients with profound BSNHL. Our results demonstrated that a homozygous mutation of *TMPRSS3* was associated with the congenital hearing loss in this Tibetan family. To our knowledge, this is the first report of a *TMPRSS3* mutation causing hearing loss in a Chinese population. *TMPRSS3: c.535G>A* is a missense mutation in exon 6 of *TMPRSS3* and leads to the substitution of Ala to Thr at amino acid 179 (p.Ala179Thr) (Figure 3). In addition, Sanger sequencing analyses of the direct relatives in this family revealed that only the two affected brothers have the homozygous mutation, while both parents are carriers of the heterozygous mutation and the unaffected sister does not carry the mutation. These results clearly showed the co-segregation of hearing loss with this mutation in this Tibetan family. Moreover, the *TMPRSS3: c.535G>A* mutation was not detected in 101 control Tibetans (data not shown), suggesting that *TMPRSS3: c.535G>A* is a disease causing mutation rather than a polymorphism. All three function prediction tools (SIFT, PolyPhen-2, and MutationTaster) suggest that this mutation affects protein function and is a disease causing mutation (Table 2). Overall, these data support that the hearing loss in the two affected brothers in this family is caused by the homozygous *TMPRSS3: c.535G>A* mutation.

After the first published report of a protease associated with hearing loss by Scott *et al.* [8], 23 *TMPRSS3* mutations have been reported as pathogenic mutations for inheritable deafness (Table 3). Among these mutations, 15 mutations are located in the serine protease domain, three in the SRCR domain, and five in the LDLRA domain. The finding of the homozygous missense mutation (p.Ala179Thr) in our patients added one more case of BSNHL caused by a mutation in the SRCR domain of TMPRSS3.

**Table 3:** Pathogenic deafness mutations in *TMPRSS3* identified in previous studies.

Ethnicity	Nucleotide	AminoAcid Change	FunctionalDomain	Type ofVariant	Reference
British	c.413C>G	p.Ala138Glu	SRCR	mis	13
Dutch	c.595G>A	p.Val199Met	SRCR	mis	13, 26
Pakistani	c.207delC	p.Thr70fs	LDLRA	del	17, 18
Spanish					
Greek					
Newfoundlander					
German	c.916G>A	p.Ala306Thr	TM	mis	12, 14,19
Korean					
Dutch	c.1276G>A	p.Ala426Thr	TM	mis	13
Turkish	c.647G>T	p.Arg216Leu	TM	mis	17
Pakistani	c.325C>T	p.Arg109Trp	LDLRA	mis	9,
Korean					
Pakistani	c.581G>T	p.Cys194Phe	SRCR	mis	9
Turkish	c.1192C>T	p.Gln398X	TM	mis	20
Tunisian	c.1211C>T	p.Pro404Leu	TM	mis	20
UK Caucasian	c.268G>A	p.Ala90Thr	LDLRA	mis	21, 22
Moroccan					
Greek	c.308A>G	p.Asp103Gly	LDLRA	mis	18
German	c.646C>T	p.Arg216Cys	TM	mis	14
Pakistani	c.1219T>C	p.Cys407Arg	TM	mis	9, 16, 17
Korean	c.743C>T	p.Thr248Met	TM	mis	12
Pakistani	c.767C>T	p.Arg256Val	TM	mis	16
Tunisian	c.753G>C	p.Trp251Cys	TM	mis	10
Pakistani	c.1273T>C	p.Cys425Arg	TM	mis	16
Pakistani	c.310G>A	p.Glu104Lys	LDLRA	mis	16
	c.310G>T	p.Glu104Stop		non	
Palestinian	c.1180_1187del8ins68	_	TM	_	8
Italian	c.1019C>G	p.Thr340Arg	TM	mis	23
Italian	c.1291C>T	p.Pro431Ser	TM	mis	23
Newfoundlander	c.782+8insT	-	TM	-	17

TM: transmembrane domain; LDLRA: low-density lipoprotein receptor A domain; SRCR: scavenger-receptor cysteine rich domain; Mis: missense; Del: delete; Non: nonsense.

**Figure 5:**
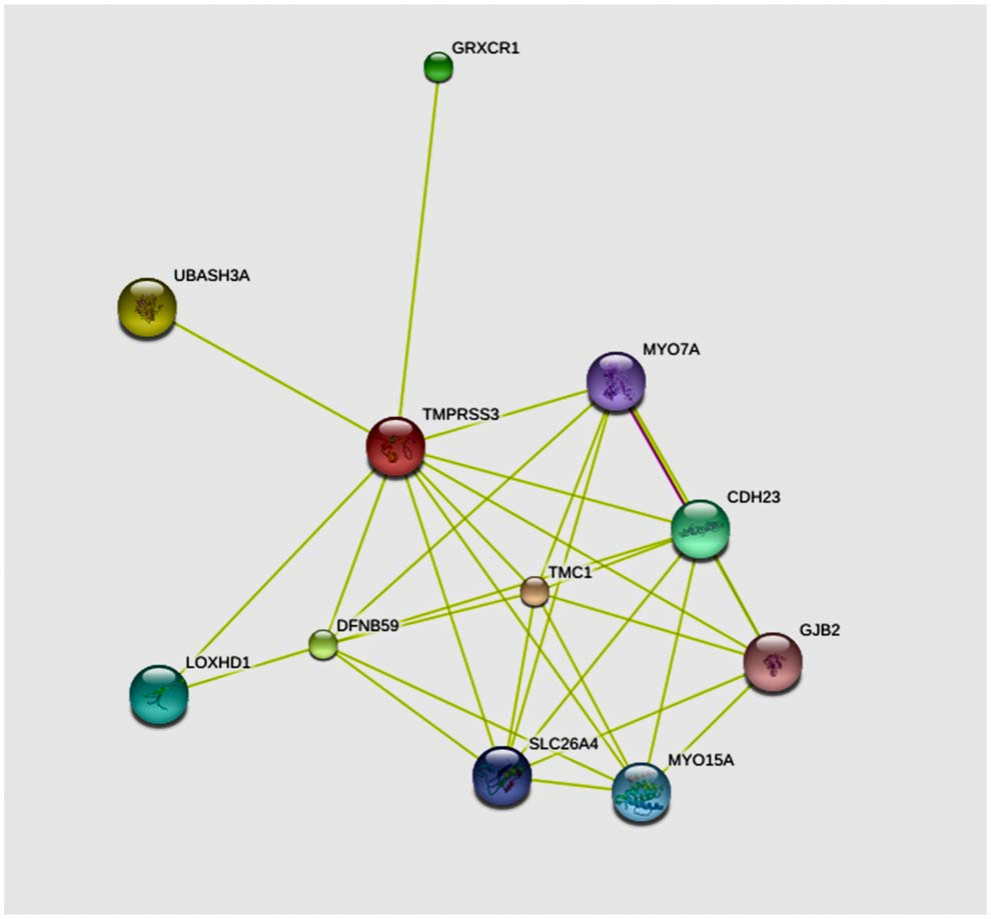
The interactive protein network for human TMPRSS3 predicted by STRING 9.1. Proteins predicted to interact with human TMPRSS3 include multiple known deafness proteins such as MYO7A, GJB2, DFNB59 and SLC26A4. The software tool is available in the website: http://string-db.org/.

Proteins containing SRCR domains have been proposed to function in the homeostasis of epithelia and the immune system and some have been reported to be associated with a number of diseases and pathogenic states such as atherosclerosis, Alzheimer’s disease, autoimmune diseases, and cancer. Thus, they exhibit promising potential as targets for diagnostic and/or therapeutic intervention [12], [13]. p.A138G, p.V199M and p.C194F are the mutations in the SRCR domain of TMPRRS3 that have been found to be associated with deafness [10], [14], [15]. The mutation found in our patients results in p.A179T in the SRCR domain of TMPRRS3. The SRCR domain includes amino acids from V110 to T205 and contains four cysteine rich motifs, which can bind negatively charged molecules such as lipoproteins and sulphate polysaccharides. Proteins that are predicted by the online software STRING 9.1 to interact with TMPRSS3 in humans include multiple known deafness proteins such as MYO7A (MIM#276903), GJB2, DFNB59 (MIM#610219), and SLC26A4 (Figure 5). Therefore, the amino acid change p.A179T in the SRCR domain in our patients likely affects the interaction of TMPRSS3 with other hearing-associated proteins, leading to hearing damage. Nevertheless, further studies with a large case volume are needed to establish the clear genotype and phenotype association for mutations in the SRCR domain.

Experiments using the Xenopus oocyte expression system conducted by Guipponi *et al.* have shown that TMPRSS3 cleaves epithelial sodium channel (ENaC) and promotes ENaC mediated currents [16]. This report also showed co-expression of ENaC and TMPRSS3 in Corti’s organ, stria vascularis and spiral ganglions, indicating that TMPRSS3 may play a role in signal transduction between spiral ganglions and hair cells. Molina *et al.* showed that TMPRSS3 in mice maintains normal expression of KCNMA1 potassium channels and normal outward K^+^ currents in inner hair cells (IHCs) [17]. The mutation identified in our study does not fall in the protease domain. However, it would be interesting to test whether this mutation indirectly interferes with protease activity and affects normal outward K^+^ currents in IHCs.

It has been shown by multiple studies that the detection rates of *TMPRSS3* mutations are variable in different ethnic groups [10], [14], [18], [19], [20]. Thus far, *TMPRSS3∶207delC* is the most commonly reported deafness mutation for this gene. It has been detected in Spanish people, Grecians, Newfoundlanders, and Pakistan populations [19], [20]. *TMPRSS3* mutations account for 5.9% of autosomal recessive non-syndromic hearing loss (arNSHL) cases and 8.3 of postlingual arNSHL cases in Korean patients [21]. The mutation *TMPRSS3∶916G>A*, which has been detected in German and Korean deaf patients, leads to an amino acid change A306T in the transmembrane domain [18], [21], [22]. This mutation results in decreased protease activity, which may explain its underlying molecular etiology for deafness. Moreover, the mutation pR109W in the LDLRA domain of TMPRSS3 has been detected in multiple Asian familiar deafness cases [10], [22]. Weegerink *et al.* reported that mutations in different domains of TMPRSS3 resulted in varied hearing loss phenotypes, likely due to the distinct influence of protease activity by various mutations [14]. Several other studies have also found a variety of *TMPRSS3* mutations in patients with hearing loss (Table 3) [11], [23]–[27]. The genetic spectrum of arNSHL in Chinese patients mainly includes mutations in *GJB2*, *SLC26A4*, *MYO7A*, *POU3F4*, *USH2A* and *TMC1*. Mutations in *GJB2*, followed by mutations in *SLC26A4*, are the most commonly identified cause of sensorineural hearing loss in Chinese patients [2]. Dai *et al.* suggested that hotspots for hearing loss mutations are variable in different regions in China [5]. Their studies found distinct allele frequencies for *GJB2∶235delC* in different regions in China. It has also been reported that some common molecular etiologies, such as *GJB2* and *SLC26A4* mutations, in the general Chinese deaf population are rare in the Tibetan Chinese deaf population [28]. Consistent with these studies, our microarray screening of nine common deafness mutations in *GJB2*, *GJB3*, *SLC26A4* and *12S rRNA* in Tibetan Chinese patients with hearing loss resulted in a low positive detection rate.

In this study, we reported the first mutation in *TMPRSS3* (*TMPRSS3: c.535G>A* in exon 6, resulting p.A179T in the SRCR domain) found in the Chinese Tibetan population. Our results provided a new example of prelingual deafness caused by a *TMPRSS3* mutation.
